# Contemporary demographic challenges and population policies

**DOI:** 10.2471/BLT.24.291641

**Published:** 2024-04-01

**Authors:** Stuart Gietel-Basten

**Affiliations:** a Division of Social Science and Division of Public Policy, The Hong Kong University of Science and Technology, Clear Water Bay, Kowloon, Hong Kong Special Administrative Region, China.

The contemporary debate regarding global population and development seems to be grounded in anxiety and fear, just as it was during the International Conference on Population and Development in Cairo, Egypt, in 1994. Then, the primary anxiety was around rapid population growth and sustainable development. Today, anxiety about population growth has been joined by that of climate change and a growing fear of population stagnation, decline and ageing.

Policy-makers are still tempted to seek demographic solutions to what they perceive as demographic problems. In some cases, access to sexual and reproductive health services are being restricted, at least in part, in the name of demographic survival. Target-based, pro- and anti-natalist policy-making restricts individual choice, directly opposes the principles of the conference, and suggests a continuing mismatch between the families that individuals aspire to build and the families they are allowed or supported to build.

Disentangling motivation from ideology is challenging because the demographic motivation of many pro-natalist policies seems ideologically grounded in a heteronormative view of the family that reduces women to a reproductive role, and excludes sexual (and other) minorities from realizing their reproductive aspirations. Comprehensive sexual education, often misunderstood and debated over, is a critical component of sexual and reproductive health,^1^^,^^2^ but implementation of these programmes is widely contested by stakeholders. 

Thirty years on, the world is a more demographically diverse place, with a wider set of population trajectories that encompass rapid growth as well as ageing and decline, and reproductive autonomy is just as pertinent today as it was then.

As in 1994, the population in many countries is still growing at a rapid pace. To at least some extent, rapid population growth reflects gaps in available, accessible and acceptable quality sexual and reproductive health services that respond to a range of needs and wishes over time.^3^ Indeed, while sexual and reproductive health services have expanded in the past 30 years, progress has been uneven, and global inequities within and between populations still characterize almost every aspect of these services.^4^ Research, policy, guidelines and implementation of sexual and reproductive health continue to overlook specific communities highlighted in the conference’s programme of action.^5^ These communities include persons with disabilities, Indigenous peoples, migrants and those forcibly displaced.^6^^–^^8^ Failures to reach these populations are especially concerning as their needs are often the greatest. 

Meanwhile, global population ageing has highlighted the importance of previously neglected issues in sexual and reproductive health. Sexual health of older people has been overlooked for many years and remains taboo in many countries. The mean age of first birth is now above 30 years in many countries, and the age-specific fertility rate among women 35–39 years of age has doubled since 1994 in parts of the European and South-east Asian regions.^9^ The increasing age of first birth has grown in tandem with demand for fertility care services. Approximately one in every six people of reproductive age worldwide experience infertility in their lifetime, but fertility care is inaccessible for most people globally.^10^

The unfinished business of the conference’s agenda is now set against new challenges. Responding to the contemporary demographic challenges will require a profound revision of the 1994 programme of action. Yet, our current demographic diversity can be leveraged to further increase the relevance and power of this narrative. Regardless of their population size, all countries are grappling with unique risks and have variable available resources. No one-size-fits-all policy exists to ensure that development is sustainable and that individuals and communities are supported to thrive in all stages of life. Nor can complex demographic challenges be solved by compelling or coercing women to have more or fewer children. 

Substantial evidence demonstrates that institutional reform, promotion of inclusivity and equity, investments in human capital, increased productivity and behaviour change are much more effective means of responding to the challenges associated with demographic change than demographic engineering.^11^^,^^12^ This evidence backs the argument that human reproduction and, by definition, sexual and reproductive health, must be decoupled from demographic, economic and political aims, reiterating the centrality of human rights and autonomy. 

Instead of focusing on numbers, population policies should focus on dignity, inclusivity, equity, gender equality, decent employment and social protection. Comprehensive quality sexual and reproductive health services and education are critical prerequisites to allow everyone to reach their full potential.
